# In Vitro and In Vivo Investigation on the Effectiveness of Alginate-Based Gastric Mucosal Protective Gel

**DOI:** 10.1155/2022/8287163

**Published:** 2022-08-24

**Authors:** Hang Zeng, Muye He, Minyi Yang, Zhu Meng, Han Wang, Chunren Wang

**Affiliations:** ^1^National Institute for Food and Drug Control, Beijing 102629, China; ^2^China Pharmaceutical University, Nanjing 211198, China

## Abstract

**Objective:**

To investigate the feasibility and effectiveness of an alginate-based gastric mucosal protective gel on the gastric ulcer.

**Methods:**

(1) In the physical protection model, after GES-1 cell attachment add the gel to transwell chamber, add different concentrations of HCl to the gel. Absorbance was measured to assess proliferation and images of the cells migrating into the wound were taken; then the migration rate of the cells was quantified by comparing images. (2) In the gastric ulcer model, excise the gastric mucosal of SD rats; the gel and fixative were applied on the artificial ulcer immediately. Dissect rats after 10 days, and calculate the wound healing rate and analyzed histology changes.

**Results:**

The effect of hydrochloric acid on cells in the lower layer was significantly reduced after the use of gastric mucosal protection gel. The protective gel had an isolation effect on different concentrations of acid. A number of GES-1 were significantly higher than those in the control group at 24 h to 72 h (*P* < 0.01). The migration was observed compared with the control group. The average healing rate of ulcer in the gel group was about 50%, and the control group was about 30%. Inflammation occurred in all wound regions after ten days. In the gel group, inflammatory infiltration depth was lower than that of the control, and part of SD rats' new muscle layer appeared without inflammatory infiltration. The connective tissue proliferation promoted tissue repair. In the control group, necrosis marginal, mucosal hyperplasia, marginal lymphocyte aggregation, and bleeding were observed.

**Conclusion:**

This novel gel mainly has an isolating and shielding effect to prevent the wound from being exposed to gastric acid for a long time, and it can reduce the inflammatory reaction on the wounds to promote the healing of the ulcer. The gastric mucosal protective gel cannot only promote the speed of wound healing but also improve the quality of wound healing.

## 1. Introduction

Gastric cancer, as a malignant tumor caused by gastric mucosal epithelial lesions, is one of the diseases of cancer-related deaths worldwide [1–9]. The treatment of early gastric cancer is particularly important for preventing the further deterioration of gastric cancer and reducing the death rate of gastric cancer [6–10]. Endoscopic submucosal dissection (ESD) is a minimally invasive surgery for the resection of early gastric cancer and benign gastric tumors [9–14].

ESD can resect a larger lesion compared with endoscopic mucosal resection (EMR) [14–17]. With the advantages of less trauma and lower recurrence rate, it has gradually replaced some traditional surgical procedures [17–19]. However, ESD usually results in a larger ulcer wound than EMR, and a high frequency of complications such as severe perforation and bleeding has been found for ESD [16, 17]. It has been reported that an ESD-induced ulcer needs 8 weeks to heal because of exposing longer to gastric acid and pepsin [12, 17, 20, 21]. In the first 4 weeks after ESD, the ulcer is in a healing state but for the first 3 days after ESD, massive bleeding usually occurs. The current treatment methods for wound repair after ESD still cannot avoid bleeding and perforation [22, 23]. Therefore, in the early phase, it is necessary to provide a product that can be directly applied to the gastric mucosal surface through gastroscopy to protect the ulcer from the attack of gastric or bile secretions [24]. The development of biomaterials provides new ideas for the development of this product.

Alginate is a natural polyanionic copolymers derived from brown sea algae [25]. In general, alginate forms stable gels via ionic interactions between carboxylic acids and divalent cations such as Ca^2+^, Mg^2+^, and Ba^2+^ [25–29]. In recent years, alginate as a novel material has been widely used in the wound repair because of its features such as biological origin, gel forming ability, biocompatibility, and biodegradability [29–32]. Several alginate-based wound dressings are now commercially available [30].

A novel biomaterial gel was designed by our team [24]. It contains a colloidal solution and a fixative solution. The main components of the colloidal solution are alginate and polylysine. The fixative solution contains calcium ions. Colloid solution and fixative solution can be directly sprayed onto the damaged gastric mucosa surface through gastric speculum, forming gel under the action of gastric acid, which can protect gastric mucosa and accelerate wound healing.

In this study, the physical protection model and gastric ulcer model aimed to evaluate the feasibility and effectiveness of the curative of the newly gastric mucosal protective gel on the ESD-induced ulcer and provided data support for its clinical application.

## 2. Materials and Methods

### 2.1. Cell Lines and Animals

Human gastric epithelial GES-1 cells were purchased from Pituo Biology Co., Ltd. (Shanghai, China).

Twenty female SD rats aged approximately 6-7weeks and weighing 250 ± 20 g were purchased from the Laboratory Animal Resource Center of National Institutes for Food and Drug Control (Beijing, China) with the Beijing Association for Science and Technology (SYXK [Beijing] 2007–0013). In the independent ventilation cages (IVC), all animals were housed at the temperature of 20-25°C and humidity of 40-70%. They were allowed access to diet and tap water ad libitum.

### 2.2. Description of the Gastric Mucosal Protective Gel

The novel gel contains colloid solution and fixative solution. The colloid solution contains mainly seaweed polysaccharide and polylysine. The fixative solution contains calcium. The two solutions can rapidly self-assemble forming a solid-film with a complex network of polysaccharides and amino acids, which further solidifies in the presence of gastric acid [24]. The gel adheres to the ulcer floor through cross-linking.

### 2.3. Physical Protection Model

**Figure 1:**
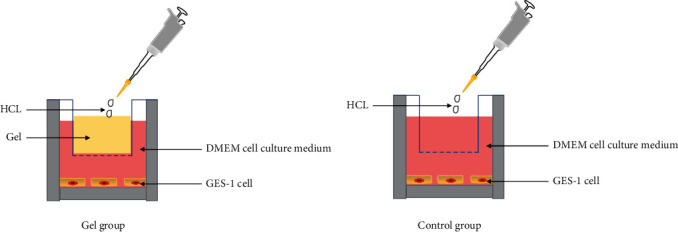
The schema of the experiment to evaluate protective effect of the gel on GES-1 cultured under hydrochloric acid treatment.

To assess the effect of Gastric mucosal protective gel on GES-1 proliferation and migration, we designed a physics-protection experiment according to the literature [33] and the characteristics of the gel (Figure 1). GES-1 cells were suspended in DMEM and seeded into each well of a transwell plate. After cell attachment, add 0.2 ml of gel to the transwell chamber. The cells were incubated with the DMEM medium and add hydrochloric acid to the gel for some hours and measure cell survival.

#### 2.3.1. Cell Proliferation Studies

Cell number was measured using the MTT method [34–36]. Briefly, the GES-1 cells in the logarithmic growth were suspended in DMEM and seeded into each well of a transwell plate at a density of 2 × 10^4^/well. After cell attachment, add 0.2 mL of gel to the transwell chamber of the sample group. The control group was not treated. The cells were incubated with the DMEM medium and add the different concentrations of concentrated hydrochloric acid (50 *μ*L1/8 HCl, 1/16 HCl, 25 *μ*L1/8 HCl, 12 *μ*L1/2 HCl, and 1/4 HCl) to the gel for 24 h, 48 h and 72 h. MTT was added and cultured for 2 h, then the Isopropanol was added. Absorbance was measured at 570 nm and 650 nm.

#### 2.3.2. In Vitro Wound Healing Assay

Cell migration was analyzed with the in vitro scratch assay [37, 38]. The GES-1 cells in the logarithmic growth were cultured in 24-well plates at a density of 6 × 10^4^/well, and after the induction of quiescence, a single scratch wound was created in the centre of the cell monolayer by the gentle removal of the attached cells with a sterile plastic pipette tip. Add 0.2 mL of gel to the transwell chamber of the sample group. The control group was not treated. The cells were incubated with the DMEM medium and add different concentrations of concentrated hydrochloric acid (50 *μ*L-1/16HCl, 25 *μ*L-1/8HCl, and 12 *μ*L-1/4HCl) to the gel for 24 h. Images of the cells migrating into the wound were taken at 0 h and then every 12 h until the scratch wound was closed at 24 h. The images were captured to evaluate the migration rate of every group. The closure of the wound was considered to represent 100% migration. The cell images were captured using a microscope (Olympus IX71, Spain) and analyzed using imaging software (ImageJ).

### 2.4. Gastric Ulcer Model

**Figure 2:**
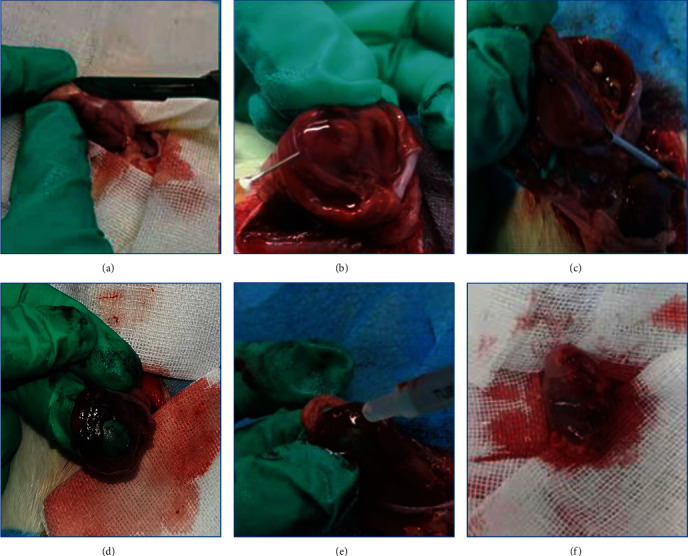
Endoscopic submucosal dissection (ESD) in rats. (a) Cutting along the greater curvature of the stomach; (b) injection of saline into the gastric mucosa layer; (c) excise the mucosa of similar size and area; (d) artificial gastric ulcer; (e) spraying gastric mucosal protective gel on the traumatic; (f) fixing with nylon sutures.

The procedures used in the animal experiments are summarized in Figure 2 [33, 39–41]. All SD rats were fasted for 24 h and then anesthetized by intramuscular injection of Zoletil 50. Then stomachs of anesthetized rats from each group were opened along the greater curvature and rinsed with normal saline (NaCl 0.9%). Expose the gastric mucosa, and injecte 0.2 mL of saline to form a bulge, then excise the mucosa of similar size and area to form a wound. The SD rat was randomized into the control group (*n* = 10) and the gel treated group (*n* = 10). The colloid solution was sprayed on the ulcer surface and then the fixative solution was sprayed. The gel solidified about 3–5 min. In the control group, the ulcer was managed routinely. Then photograph and suture the stomach.

#### 2.4.1. Evaluation of Artificial Ulcer Healing

Dissect rats after 10 days, and remove the gastric ulcer wound. The percent of healing areas was calculated with dividing healing part area by the area of originally resected tissue. The percent remaining area of the artificial ulcer was calculated by dividing the ulcer area by the area of initially resected tissue. The final percent healing area was defined as 100% percent remaining area described earlier.

#### 2.4.2. Histological Screening of Gastric Mucosa

Dissect rats after 10 days, and remove the gastric ulcer wound and fix in 10% neutral buffered formalin [42, 43]. All wounds including the ulcer and near to usual tissue were taken to perform the histology examination. Stomachs were flushed with PBS (pH 7.4) and fixed in 4% paraformaldehyde in 0.1 M phosphate buffer. Fixed specimens were processed using the conventional paraffin embedding technique including dehydration through ascending grades of ethanol and clearing in 3 changes of xylene and melted paraffin and ended by embedding in paraffin wax at 65°C. Paraffin blocks were sectioned into 4 *μ*m thickness sections. These sections were stained by hematoxylin and eosin (HE) staining according to the method described by Bancroft and Layton. Then, the histopathological morphology of the general microstructure of gastric mucosa was observed under a digital camera (Leica EC3, Leica, Germany) connected to a microscope (Leica DM500).

#### 2.4.3. Immunohistochemical Examination of CD34 Proteins

Angiogenesis was evaluated by CD34 immunohistochemistry [44]. Briefly, 4 *μ*m thick paraffin sections were prepared and deparaffinized using xylene, rehydrated in graded alcohols, and finally washed with distilled water. Antigen retrieval was done in the case of anti-CD3 by heating in 10 mM citrate buffer (pH 6.0) for 10 min at 95°C. Deactivation of endogenous peroxidase was carried out using 3% H_2_O_2_ in absolute methanol for 10 min at room temperature. After washing with PBS, the nonspecific reaction was blocked with goat blocking serum for 30 min at room temperature. The sections were incubated at 4°C overnight with the specific primary antibody: polyclonal rabbit anti-human CD3 antibody (Abcam, Cat: ab5690). After washing with PBS, the paraffin section was incubated by biotin-conjugated goat anti-rabbit IgG antiserum (Histofine kit, Nichirei Corp.) for 45 min and then washed with PBS. The streptavidin-biotin complex was visualized with 3,3′-diaminobenzidine tetrahydrochloride (DAB) H_2_O_2_ solution, pH 7.0, for 5 min. Then sections were washed in PBS and Mayer's hematoxylin was used as a counterstain. The sections were washed in distilled water; then use ethanol gradient dehydration and soak in xylene for 5 minutes for transparent and neutral resin for mounting. The section images were taken with a digital camera (Leica EC3, Leica, Germany) connected to a microscope (Leica DM500).

### 2.5. Statistical Analysis

The results were represented as the means ± SD of at least 3 separate experiments. A *t*-test or a one-way analysis of variance was used to analyze differences between the means, which was followed by a Dunnett post hoc test for multiple comparisons. The differences were deemed to be obvious at *P* < 0.05. The statistical analyses were calculated by the IBM SPSS Statistics 26 software.

## 3. Results

### 3.1. Physical Protection Model

#### 3.1.1. Evaluation of Protective Effect of the Gel on Human Gastric Epithelial Cell (GES-1) Proliferation under Hydrochloric Acid Treatment

200 *μ*L of gastric mucosal protective gel had an isolation effect on different volumes and concentrations of hydrochloric acid. The adjustment of diluted hydrochloric acid was significantly more obvious than those in the undiluted hydrochloric acid group.

**Figure 3:**
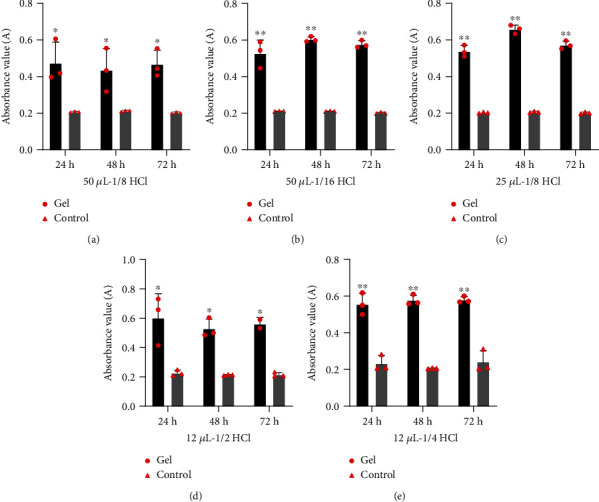
The result of experiment to evaluate protective effect of the gel on GES-1 cultured under hydrochloric acid treatment. (a–e) The absorbance of the different volumes and concentrations of hydrochloric acid (50 *μ*L1/8HCl, 1/16HCl, 25 *μ*L1/8HCl, 12 *μ*L1/2HCl, and 1/4HCl) in gel and control group for 24 h to 72 h. ^∗^*P* < 0.05 to control; ^∗∗^*P* < 0.01 to control.

As shown in Figure 3, the number of GES-1 in the gel group was significantly higher than those in the control group at 24 h to 72 h. Then, three groups of hydrochloric acid concentrations (50 *μ*L-1/16 group, 25 *μ*L-1/8 group, and 12 *μ*L-1/4 group) were selected with significant differences (*P* < 0.01) in proliferation results to continue the wound healing assay in vitro.

#### 3.1.2. Evaluation of Protective Effect of the Gel on Human Gastric Epithelial Cell (GES-1) Migration under Hydrochloric Acid Treatment

**Figure 4:**
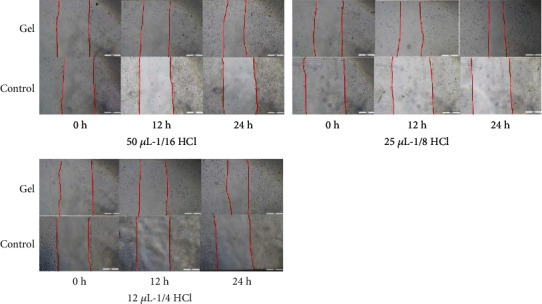
The cell migration of different concentrations of hydrochloric acid group.

To address the effect of the gel on GES-1 migration, a wound healing assay was performed. We analyzed the cell migration every 12 h for 24 h. As shown in Table 1, the gel protected the migrated GES-1 and the cells in the control group did not migrate at 12 h and 24 h (Figure 4).

### 3.2. Gastric Ulcer Model

#### 3.2.1. Wound Healing Rate

**Figure 5:**
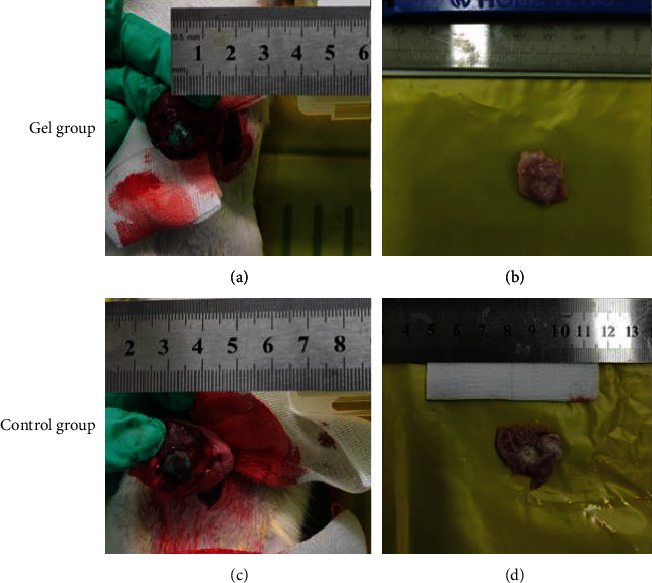
The ulcer lesions on the ten days. (a) Artificial gastric ulcer of gel group; (b) the ulcer healing of ten days of gel group; (c) artificial gastric ulcer of control group; (d) the ulcer healing of ten days of control group.

**Figure 6:**
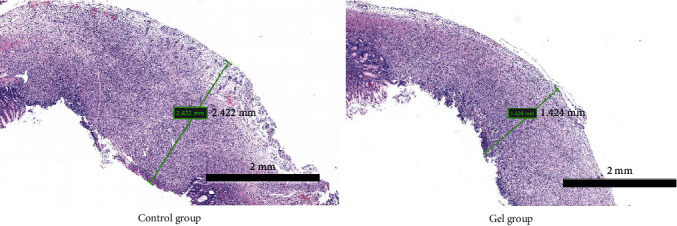
Wound repair and inflammatory infiltration (5x).

Ten days after the operation, all rats in the gel group and the control group were in good condition. All rats were sacrificed with CO_2_ euthanasian and were observed the wounds of gastric ulcer. Two rats in the control group had hemorrhage on the ulcer surface, but there was no significantly blood in the gel group (Figures 5 and 6).

**Figure 7:**
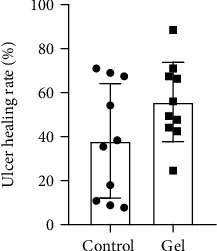
Percent healing area ratio of gastric ulcer.

Statistics of wound area showed that the average healing rate of ulcer in the control group was about 30%, and the healing rate of the gastric mucosal protective gel group was about 50%. The overall healing rate of the protective gel group was better than that of the control group (Figure 7).

#### 3.2.2. HE Staining

**Figure 8:**
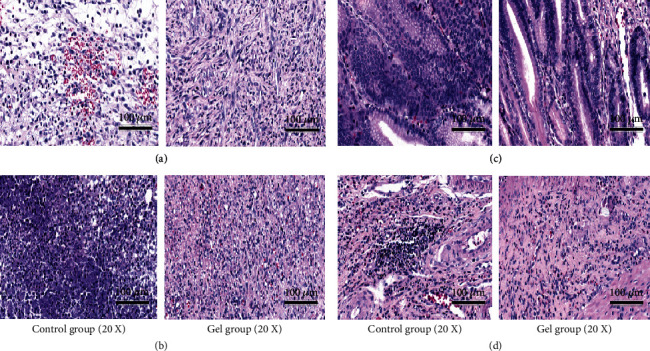
The histopathological morphology of gastric mucosa after 10 days. (a, b) A large number of connective tissues were found proliferation in the gel group, but in the control group, there was found extensive necrosis and occurred edema and bleeding; (c, d) in the gel group, the marginal mucosal epithelial cells arranged regularly and there was no proliferation but in the control group, marginal mucosal epithelial cells had irregular proliferation, and a large number of lymphocytes in the marginal muscle layer aggregated (20x).

Inflammation occurred in the basal layer of the gastric mucosal in all wound regions after ten days, and the number of the gel group was significantly higher than the control group (*P* < 0.01). There was a large number of connective tissue proliferation to promote tissue repair. The control group showed extensive necrosis and occurred edema and bleeding, marginal mucosal epithelial cells had irregular proliferation, and a large number of lymphocytes in the marginal muscle layer aggregated. The gel group was no necrosis and the marginal mucosal epithelial cells arranged regularly, and there was no proliferation. Inflammatory infiltration depth of the gel group was lower than the control, and there was no significant difference in inflammatory infiltration depth. Part of the experimental group rats appeared new muscle layer without inflammatory infiltration (Figure 8).

#### 3.2.3. Immunohistochemistry of CD34

**Figure 9:**
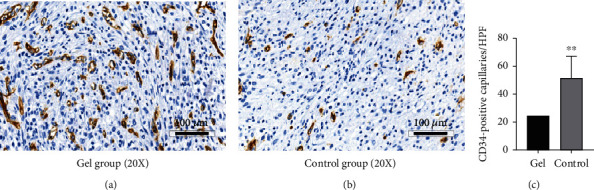
The immunohistochemistry of CD34(+) after 10 days. (a) CD34 immunohistochemistry of gel group (20x); (b) CD34 immunohistochemistry of control group (20x); (c) number of stained blood vessels of gel and control groups. The difference between the control and the gel groups was statistically significant. ^∗∗^*P* < 0.01 to control.

There was an increasing in the proportion of CD34(+)-perfused vessels in all wound regions after ten days, and the number of the gel group was significantly higher than that of the control group (Figure 9).

## 4. Discussion

Endoscopic gastric mucosal resection and gastric mucosal dissection are minimally invasive procedures for the removal of early gastric cancer and benign gastric tumors. It not only achieves the purpose of radical treatment of early gastric cancer but also has the advantages of less trauma and less impact on the quality of life of patients, which has gradually replaced some traditional surgical procedures [8–10, 17–19]. However, there is no good measures to protect the wound after the surgery at present, and the wound after mucosal dissection is only treated with hemostasis, which is not conducive to wound healing. Under the action of gastric acid and pepsin, the wound healing time is longer, and the wound is easier to form scar tissue [16, 17]. Clinical research [45] showed that the healing rate of patients undergoing endoscopic gastric mucosal resection and gastric mucosal dissection at 6 weeks was about 69%. Therefore, it is necessary to provide a product that can be applied directly to the mucosal surface of gastric injury through gastroscopy. At present, the dominant wound treatments for endoscopic submucosal dissection include endoscopic closure and drug therapy, but the results are not ideal. The exposed wounds tend to cause delayed bleeding and perforation. Therefore, it is of great clinical value in the recovery of gastric ulcers and wounds after endoscopic submucosal dissection to study the key technologies of regenerative materials for gastric mucosal wound repair, develop new regenerative medical biomaterial technology products, and expand new markets.

Gastric mucosal wound protective gel includes a colloidal solution and a fixative solution. The colloidal solution is mainly composed of sodium alginate and polylysine, and the fixative solution is a cationic compound solution [24]. The alkaline polylysine in colloid solution is electrically neutral and not cross-linked with sodium alginate. The colloid solution is sprayed by the gastroscope to the gastric mucosal; then, the fixation is sprayed so that the cations in the fixation and the sodium alginate in the colloidal solution cross-link, to solidify and avoid flow. In the colloid solidification and acidic conditions in the stomach, the negatively charged sodium alginate and the positively charged polylysine (in the acidic conditions polylysine is positively charged) are further cross-linked through the positive and negative electric attraction. When the colloid liquid becomes a gel film, the positive charged polylysine will also adhere to the gastric mucosa (cells with negative charge), so that the protective gel can be tightly adhered to the wound surface. The solidified protective gel can physically isolate the mucosa from gastric acid and pepsin to protect the gastric mucosa, promote mucosal regeneration and accelerate wound healing. After 1 to 2 weeks, the mucosal wound heals and the protective gel is shed and exited along the intestine.

In this study, the effect of hydrochloric acid on cells in the lower layer was significantly reduced after the use of gastric mucosal protection gel in the in vitro cell culture assay, compared to the direct effect of hydrochloric acid. The reason was that the protective gel was alkaline. On the one hand, it could neutralize gastric acid, and on the other hand, it could block the diffusion of gastric acid, thus acting as an isolation barrier. In animal experiments, the protective gel group and the control group had certain promoting effect on wound healing. In the control group, 30% of the animals had a wound healing rate of less than 20%, whereas in the protective gel group all healing rates were above 20%. In the protective gel group, 90% of the animals had a wound healing rate of more than 40%, while that of only 40% animals in the control group had a wound healing rate of more than 40%. Therefore, it can be considered that the healing effect of the protective gel group is better than that of the control group. Histopathological studies revealed that many animals in the control group showed necrosis, bleeding, and severe inflammatory reaction. However, in the experimental group, the inflammatory reaction was mild, the granulation tissue was in good condition, and the regenerated epithelium of the gastric mucosa was well aligned. And immunohistochemical staining also showed that capillaries increased significantly compared with the control group. These could indicate the protective gel has a good protective effect on the wounds of gastric mucosa. Therefore, the gastric mucosal protective gel mainly has an isolating and shielding effect. It reduces the inflammatory reaction on the wounds, promotes the establishment of granulation tissue, especially capillary microcirculation of new tissue, and promotes the regeneration of the gastric mucosal endothelium. The gastric mucosal protective gel cannot only promote the speed of wound healing but also improve the quality of wound healing. As early as 1991, Tarnawski et al. [46] found abnormal thickness of the regenerating mucosa and submucosal vascular network in the study of peptic ulcer and proposed the concept of “ulcer healing quality” at the histological and molecular levels. They pointed out that healing of ulcers requires not only the repair of surface mucosa but also the repair and reconstruction of submucosal tissue structures. In their study, they found although the epithelium in the ulcer initial healing was intact, the tissue structure was obviously abnormal. The abnormalities reduced the cell oxygenation ability, energy supply, and mucosal defense function, so they suggested that the poor quality of the ulcer healing was the pathological basis of ulcer recurrence. The main indicators of the quality of healing of gastric ulcer mucosal were the maturity of mucosal structures and the maturity of the submucosal vascular network. In our study, it was found that the regenerated mucosal epithelium was orderly arranged and capillaries were abundant in the regenerated tissues with using of gastric mucosal protective gel. Therefore, the gastric mucosal protective gel could improve the healing quality of gastric mucosa.

Xu et al. [47] used bioadhesive hydrogel to study wound healing of porcine ulcer. The study showed that hydrogel could inhibit inflammatory response and promote regeneration of mucosal endothelial cells and angiogenesis to protect the ulcers. He et al. [48] developed a pH-responsive self-healing hydrogel and investigated the promoting healing effect of this hydrogel on the porcine gastric ulcer model. The study showed that this hydrogel had an obvious hemostatic effect in vivo and could promote the generation of type I collagen and blood vessels in the ulcers. Zhao et al. [49] developed a polyurethane intestinal submucosal matrix hydrogel and investigated the effects in a gastric ulcer model of dogs. The results showed that the polyurethane intestinal submucosal matrix hydrogel mainly accelerated wound healing by reducing the inflammatory response and promoting mucosal endothelial cell regeneration. Li et al. [24] investigated a novel self-assembled hydrogel using an artificial ulcer model of gastric surgery in pigs. The results showed that the hydrogel could promote wound healing by promoting gastric mucosal endothelial hyperplasia.

According to the literature research data and our research results, it was found that the mechanism of gastric mucosal protective gel promoting wound healing was basically consistent with the literature reports. Gastric mucosal wound protective gel forms a protective film on the wound surface, to isolate the irritation and damage of gastric acid and digestive enzymes. It promotes the wound healing and improves the healing quality mainly through three effects. One is to reduce the inflammatory response of the wound tissue. The second is to promote the tissue regeneration and the establishment of microcirculation. The third is to promote the regeneration of traumatic mucosal endothelium.

## 5. Conclusion

In conclusion, this study strongly demonstrates the feasibility and effectiveness of the novel biomaterial gel in repairing ulcer after ESD. The novel gel mainly has an isolating and shielding effect to prevent the wound from being exposed to gastric acid for a long time and it can reduce the inflammatory reaction on the wounds to promote the healing of the ulcer. The gastric mucosal protective gel cannot only promote the speed of wound healing but also improve the quality of wound healing. Further clinical trials are needed to validate its clinical utility use.

## Figures and Tables

**Table 1:** The cell migration rate of different concentrations of hydrochloric acid group.

Migration rate (%)	50 *μ*L-1/16		25 *μ*L-1/8		12 *μ*L-1/4	
	Gel	Control	Gel	Control	Gel	Control
12 h	15.68% ± 0.01	0%	21.08% ± 0.01	0%	13.73% ± 0.01	0%
24 h	33.59% ± 0.01	0%	40.29% ± 0.03	0%	29.73% ± 0.02	0%

## Data Availability

All data used to support the findings of this study are included within the article.

## Supplementary Materials

Supplementary Materials This is a graphical abstract of this study. The gastric mucosal protective gel can rapidly self-assemble forming a solid film in in the presence of gastric acid. In this study, we evaluate the feasibility and effectiveness of the curative of the newly gastric mucosal protective gel on the ESD-induced ulcer in the physical protection model and gastric ulcer model. We found the gastric mucosal protective gel can promote the speed of wound healing and improve the quality of wound healing.

## References

[1] Jeon H. K., Kim G. H., Lee B. E. (2018). Long-term outcome of endoscopic submucosal dissection is comparable to that of surgery for early gastric cancer: a propensity-matched analysis. *Gastric Cancer*.

[2] Yang L., Zheng R., Wang N. (2018). Incidence and mortality of stomach cancer in China, 2014. *Chinese Journal of Cancer Research*.

[3] Maurizio Degiuli M. S., Ponti A., Soldati T., Danese F., Calvo F. (1998). Morbidity and mortality after D2 gastrectomy for gastric cancer: results of the Italian gastric cancer study group prospective multicenter surgical study. *Journal of Clinical Oncology*.

[4] Shizuyo I., Hiroshi H., Chisato N. (1999). Evaluation of a screening program on reduction of gastric cancer mortality in Japan: preliminary results from a cohort study. *Preventive Medicine*.

[5] Bray F., Ferlay J., Soerjomataram I., Siegel R. L., Torre L. A., Jemal A. (2018). Global cancer statistics 2018: GLOBOCAN estimates of incidence and mortality worldwide for 36 cancers in 185 countries. *CA: a Cancer Journal for Clinicians*.

[6] Ferlay J., Colombet M., Soerjomataram I. (2019). Estimating the global cancer incidence and mortality in 2018: GLOBOCAN sources and methods. *International Journal of Cancer*.

[7] Chen W., Xia C., Zheng R. (2019). Disparities by province, age, and sex in site-specific cancer burden attributable to 23 potentially modifiable risk factors in China: a comparative risk assessment. *The Lancet Global Health*.

[8] Li K., Zhang A., Li X., Zhang H., Zhao L. (2021). Advances in clinical immunotherapy for gastric cancer. *Biochimica Et Biophysica Acta. Reviews on Cancer*.

[9] Qin Y., Tong X., Fan J. (2021). Global burden and trends in incidence, mortality, and disability of stomach cancer from 1990 to 2017. *Clinical and Translational Gastroenterology*.

[10] Choi J. H., Kim E. S., Lee Y. J. (2015). Comparison of quality of life and worry of cancer recurrence between endoscopic and surgical treatment for early gastric cancer. *Gastrointestinal Endoscopy*.

[11] Hoteya S., Iizuka T., Kikuchi D., Yahagi N. (2010). Clinical advantages of endoscopic submucosal dissection for gastric cancers in remnant stomach surpass conventional endoscopic mucosal resection. *Digestive Endoscopy*.

[12] Shimura T., Sasaki M., Kataoka H. (2007). Advantages of endoscopic submucosal dissection over conventional endoscopic mucosal resection. *Journal of Gastroenterology and Hepatology*.

[13] Chen X., Nishiguchi A., Taguchi T. (2020). Adhesive submucosal injection material based on the nonanal group-modified poly(vinyl alcohol)*α*-cyclodextrin inclusion complex for endoscopic submucosal dissection. *ACS Applied Bio Materials*.

[14] Girotra M., Triadafilopoulos G., Friedland S. (2018). Utility and performance characteristics of a novel submucosal injection agent (EleviewTM) for endoscopic mucosal resection and endoscopic submucosal dissection. *Translational gastroenterology and hepatology*.

[15] Yoshikawa T., Kashima H., Toyoda F. (2021). A rare case of Epstein-Barr virus-positive early gastric carcinoma with lymphoid stroma successfully treated by endoscopic submucosal dissection alone. *Clinical Journal of Gastroenterology*.

[16] Ota K., Takeuchi T., Kojima Y. (2021). Outcomes of endoscopic submucosal dissection for gastroesophageal reflux disease (ESD-G) for medication-refractory gastroesophageal reflux disease: 35 cases underwent ESD-G including 15 cases followed more than 5 years. *BMC Gastroenterology*.

[17] Urakawa S., Momose K., Hirashita T., Lowenfeld L., Milsom J. W. (2021). Endoscopic submucosal dissection of large polyps in the right colon using an endoscopic snare with a double-balloon endolumenal interventional platform: an ex vivo study in a porcine colorectal model. *Surgical Endoscopy*.

[18] Landin M. D., Guerron A. D. (2020). Endoscopic mucosal resection and endoscopic submucosal dissection. *The Surgical Clinics of North America*.

[19] Chung I. K., Lee J. H., Lee S. H. (2009). Therapeutic outcomes in 1000 cases of endoscopic submucosal dissection for early gastric neoplasms: Korean ESD Study Group multicenter study. *Gastrointestinal Endoscopy*.

[20] Goto O., Fujishiro M., Kodashima S. (2011). Short-term healing process of artificial ulcers after gastric endoscopic submucosal dissection. *Gut Liver*.

[21] Sun P., Zheng T., Hu C., Gao T., Ding X. (2021). Comparison of endoscopic therapies for rectal neuroendocrine tumors: endoscopic submucosal dissection with myectomy versus endoscopic submucosal dissection. *Surgical Endoscopy*.

[22] Libanio D., Costa M. N., Pimentel-Nunes P., Dinis-Ribeiro M. (2016). Risk factors for bleeding after gastric endoscopic submucosal dissection: a systematic review and meta-analysis. *Gastrointestinal Endoscopy*.

[23] Lim J. H., Kim S. G., Choi J., Im J. P., Kim J. S., Jung H. C. (2015). Risk factors of delayed ulcer healing after gastric endoscopic submucosal dissection. *Surgical Endoscopy*.

[24] Li M., Jin H., Shi C., Lyu B., Ying X., Shi Y. (2021). A novel self-assembled gel for gastric endoscopic submucosal dissection-induced ulcer: a preclinical study in a porcine model. *Frontiers in Pharmacology*.

[25] Li Z., Zhang M. (2005). Chitosan–alginate as scaffolding material for cartilage tissue engineering. *Journal of Biomedical Materials Research. Part A*.

[26] Lee C., Shin J., Lee J. S. (2013). Bioinspired, calcium-free alginate hydrogels with tunable physical and mechanical properties and improved biocompatibility. *Biomacromolecules*.

[27] Huang G. Y., Zhou L. H., Zhang Q. C. (2011). Microfluidic hydrogels for tissue engineering. *Biofabrication*.

[28] Seliktar D. (2012). Designing cell-compatible hydrogels for biomedical applications. *Science*.

[29] Jeon O., Bouhadir K. H., Mansour J. M., Alsberg E. (2009). Photocrosslinked alginate hydrogels with tunable biodegradation rates and mechanical properties. *Biomaterials*.

[30] Coskun G., Karaca E., Ozyurtlu M., Ozbek S., Yermezler A., Cavusoglu I. (2014). Histological evaluation of wound healing performance of electrospun poly(vinyl alcohol)/sodium alginate as wound dressing in vivo. *Bio-medical Materials and Engineering*.

[31] Alsberg E., Anderson K. W., Albeiruti A., Franceschi R. T., Mooney D. J. (2001). Cell-interactive alginate hydrogels for bone tissue engineering. *Journal of Dental Research*.

[32] Atala A., Kim W., Paige K. T., Vacanti C. A., Retik A. B. (1994). Endoscopic treatment of vesicoureteral reflux with a chondrocyte-alginate suspension. *Journal of Urology*.

[33] Hiroyuki T., Kohki Y., Hiroe M. (2016). A basic study of the effect of the shielding method with polyglycolic acid fabric and fibrin glue after endoscopic submucosal dissection. *Endoscopy International Open*.

[34] Bufalo M. C., Candeias J. M., Sousa J. P., Bastos J. K., Sforcin J. M. (2010). In vitro cytotoxic activity of Baccharis dracunculifolia and propolis against HEp-2 cells. *Natural Product Research*.

[35] Zhang W., Torabinejad M., Li Y. (2003). Evaluation of cytotoxicity of MTAD using the MTT-tetrazolium method. *Journal of Endodontia*.

[36] Akihiro Takahashi T. T., Fujioka Y., Ishikawab Y., Yokoyama M. (1996). Effects of lipoprotein(a) and low density lipoprotein on growth of mitogen- stimulated human umbilical vein endothelial cells. *Atherosclerosis*.

[37] Girona J., Rosales R., Plana N., Saavedra P., Masana L., Vallve J. C. (2013). FABP4 induces vascular smooth muscle cell proliferation and migration through a MAPK-dependent pathway. *PLoS One*.

[38] Oda A., Taniguchi T., Yokoyama M. (2001). Leptin stimulates rat aortic smooth muscle cell proliferation and migration. *Medical Science*.

[39] Kwon C. I., Kim G., Ko K. H. (2015). Bio-sheet graft therapy for artificial gastric ulcer after endoscopic submucosal dissection: an animal feasibility study. *Gastrointestinal Endoscopy*.

[40] Nishiguchi A., Sasaki F., Maeda H., Kabayama M., Ido A., Taguchi T. (2019). Multifunctional hydrophobized microparticles for accelerated wound healing after endoscopic submucosal dissection. *Small*.

[41] Takao T., Takegawa Y., Ono H. (2017). A novel and effective delivery method for polyglycolic acid sheets to post-endoscopic submucosal dissection ulcers. *Endoscopy*.

[42] Lebda M. A., El-Far A. H., Noreldin A. E., Elewa Y. H. A., Al Jaouni S. K., Mousa S. A. (2018). Protective effects of Miswak (Salvadora persica) against experimentally induced gastric ulcers in rats. *Oxidative Medicine and Cellular Longevity*.

[43] Chen W., Wu D., Jin Y. (2020). Pre-protective effect of polysaccharides purified from _Hericium erinaceus_ against ethanol-induced gastric mucosal injury in rats. *International Journal of Biological Macromolecules*.

[44] Kimura H., Nakajima T., Kagawa K. (1998). Angiogenesis in hepatocellular carcinoma as evaluated by CD34 immunohistochemistry. *Liver*.

[45] Shuyi S., Wenjie J., Kaixue R., Yongjun L. (2016). Analysis of the influencing factors of ulcer healing after gastric ESD. *Journal of Nongken Medicine*.

[46] Cui C., Chunlian W. (2014). Research progress on the quality of ulcer healing. *Journal of Clinical Research*.

[47] Xu X., Xia X., Zhang K. (2020). Bioadhesive hydrogels demonstrating pH-independent and ultrafast gelation promote gastric ulcer healing in pigs. *Science Translational Medicine*.

[48] He J., Zhang Z., Yang Y. (2021). Injectable self-healing adhesive pH-responsive hydrogels accelerate gastric hemostasis and wound healing. *Nano-Micro Letters*.

[49] Zhao L. M., Gong M., Wang R. (2021). Accelerating ESD-induced gastric ulcer healing using a pH-responsive polyurethane/small intestinal submucosa hydrogel delivered by endoscopic catheter. *Regenerative biomateria*.

