# Supplementary figures and images for: In Vitro and In Vivo Investigation on the Effectiveness of Alginate-Based Gastric Mucosal Protective Gel
Source: Biomed Res Int. 2022 Aug 24;2022:8287163. doi: 10.1155/2022/8287163 (PMC9433266; doi:10.1155/2022/8287163)

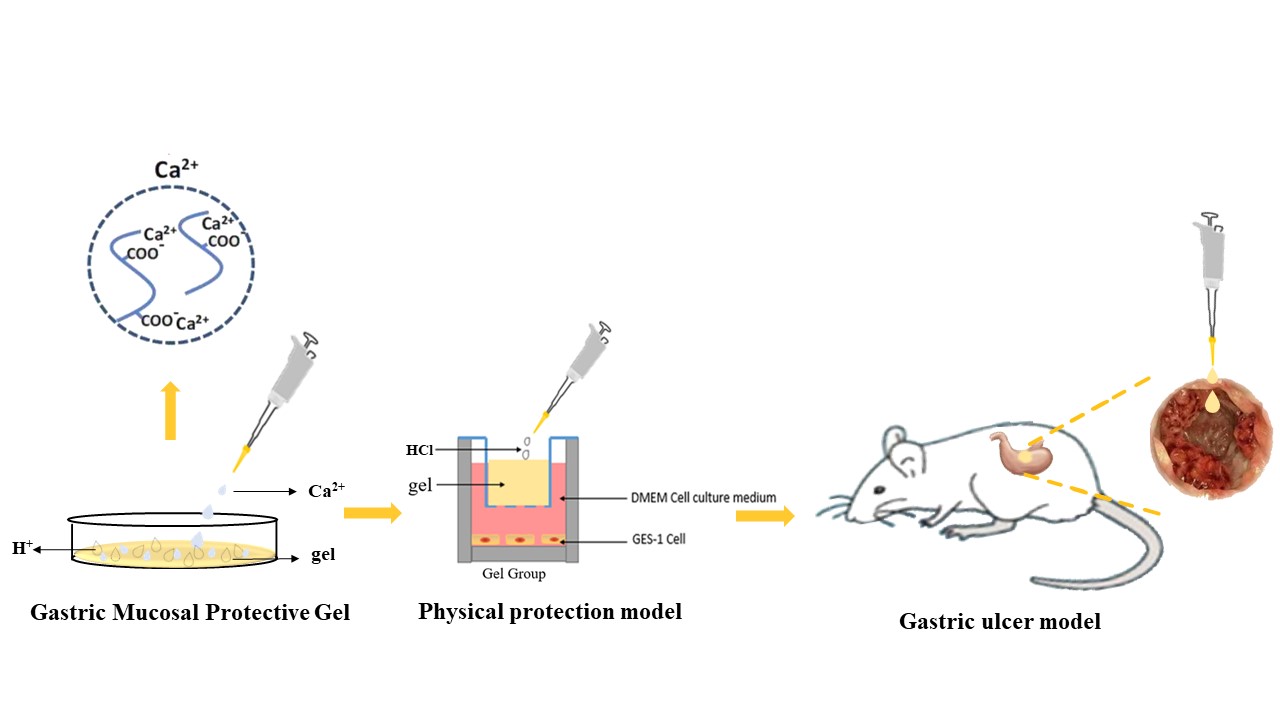

Supplement: Supplementary Materials — This is a graphical abstract of this study. The gastric mucosal protective gel can rapidly self-assemble forming a solid film in in the presence of gastric acid. In this study, we evaluate the feasibility and effectiveness of the curative of the newly gastric mucosal protective gel on the ESD-induced ulcer in the physical protection model and gastric ulcer model. We found the gastric mucosal protective gel can promote the speed of wound healing and improve the quality of wound healing. [file 8287163.f1.docx]
